# Characteristic mega-basin water storage behavior using GRACE

**DOI:** 10.1002/wrcr.20264

**Published:** 2013-06-10

**Authors:** J T Reager, James S Famiglietti

**Affiliations:** 1 Department of Earth System Science, University of CaliforniaIrvine, California, USA; 2 UC Center for Hydrologic Modeling, University of CaliforniaIrvine, California, USA

**Keywords:** GRACE, global hydrology, storage, model, remote sensing

## Abstract

[1] A long-standing challenge for hydrologists has been a lack of observational data on global-scale basin hydrological behavior. With observations from NASA’s Gravity Recovery and Climate Experiment (GRACE) mission, hydrologists are now able to study terrestrial water storage for large river basins (>200,000 km^2^), with monthly time resolution. Here we provide results of a time series model of basin-averaged GRACE terrestrial water storage anomaly and Global Precipitation Climatology Project precipitation for the world’s largest basins. We address the short (10 year) length of the GRACE record by adopting a parametric spectral method to calculate frequency-domain transfer functions of storage response to precipitation forcing and then generalize these transfer functions based on large-scale basin characteristics, such as percent forest cover and basin temperature. Among the parameters tested, results show that temperature, soil water-holding capacity, and percent forest cover are important controls on relative storage variability, while basin area and mean terrain slope are less important. The derived empirical relationships were accurate (0.54 ≤ *E_f_* ≤ 0.84) in modeling global-scale water storage anomaly time series for the study basins using only precipitation, average basin temperature, and two land-surface variables, offering the potential for synthesis of basin storage time series beyond the GRACE observational period. Such an approach could be applied toward gap filling between current and future GRACE missions and for predicting basin storage given predictions of future precipitation.

## 1. Introduction and Background

[2] The terrestrial water balance describes the partitioning of precipitation (*P*) into evapotranspiration (*ET*) and runoff (*R*). It is commonly expressed as


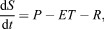
(1)

where the term d*S*/d*t* represents the change in water storage in an explicit region or control volume.

[3] Historically, various models and assumptions have been postulated to facilitate an operational relationship between precipitation forcing and runoff response within large hydrological basins. However, there is still little understanding of the primary state variable in models—terrestrial water storage—and the hydrology components of many global models are calibrated based on parameter optimization with discharge data alone [*Bonan et al*., 6; *Liang et al*., 27]. Quantitative observations of basin storage behavior are a key tool in dissecting the “black box,” in what has often been treated as an input-output relationship. However, field campaigns for storage observations are rare and never over a global domain, and the heterogeneity of land-surface properties and the complexity of land-atmosphere coupling introduce a tremendous potential for error in moving from the observational scale to the resolution of current climate models.

[4] To fulfill the objective of operational prediction skill, empirical conceptualizations of basin behavior have been necessary. Many traditional analyses have assumed that at longer timescales and over large regions, d*S*/d*t* in equation (1) can be approximated as zero. For example, *Budyko*’s [7] characterization of the relationship between catchment water balance terms offers a simple framework for understanding basin behavior. On timescales longer than annual, Budyko assumes d*S*/d*t* = 0, reducing equation (1) to *P* = *ET* + *R*. The resulting relationship, known as the Budyko curve, partitions precipitation between runoff and evapotranspiration based on the relative “dryness” of the basin. Considerable work has been done to explain deviations around this conceptual model, attributing error to variability and seasonality in climate, soil characteristics, vegetation type, and the scale of study [*Donohue et al*., 14; *Farmer et al*., 22; *Atkinson et al*., 2; *Milly*, 31].

[5] As our ability to observe large-scale Earth processes improves, we can show that approaches such as *Budyko*’s [7] formulation have important limitations. For example, terrestrial water storage *S*(*t*) has a significant interannual signal regionally, as shown by recent remote sensing campaigns [*Chen et al*., 8; *Leblanc et al*., 25; *Ramillien et al*., 35; *Syed et al*., 45; *Rodell et al*., 37; *Syed et al*., 44]. Interannual water storage signals can be caused by interannual temperature and precipitation variability, or by direct human activity [*Famiglietti et al*., 21; *Rodell et al*., 38]. This fact undoubtedly has implications for climate, ecology, and water resources availability at multiyear timescales, and natural storage processes need to be quantified based on available observations.

[6] Since terrestrial water storage in large basins can vary at interannual timescales, we might infer that these storage fluctuations are induced primarily by interannual precipitation variability. However, because of regional differences in land-surface properties, different hydrologic basins have a greater or lesser ability to buffer the effects of interannual signals in precipitation, as the land surface affects infiltration, runoff, and evaporation [*Cherkauer and Lettenmaier*, 9; *Milly and Dunne*, 1994]. Also, there are likely to be upper limits on basin storage and regional storage capacities that, when exceeded, may be linked to regional flooding [*Reager and Famiglietti*, 36; *Crowley et al*., 10].

[7] For instance, in large basins, vegetation can influence precipitation patterns by affecting moisture and energy fluxes between the surface and atmosphere [*Spracken et al*., 2012; *Bonan*, 5]. When forests decline, evapotranspiration of moisture from soil and vegetation can be diminished, leading to reduced atmospheric humidity and potentially suppressing precipitation [*Eltahir*, 17; *Eltahir and Bras*, 17; *Shukla and Mintz*, 39]. In contrast, observational studies from within regional catchments (>1000 km^2^) such as those in *Peel et al*. [34] have concluded that vegetation has a negligible impact on water balance. The discrepancy in these studies hints at the difficulties in scaling observed hydrological behavior for a global-scale understanding of the land surface and climate.

[8] The world’s largest river basins (those with an area >200,000 km^2^) account for the majority of global land runoff to the oceans [*Dai and Trenberth*, 11]. Because of the large influence of these basins on the Earth system, it is critical to work toward an accurate representation of their behavior in global climate models. For modern land-surface models operating at 2.5° or lower resolution, or to capture the major dynamics of the global water cycle, the results of traditional catchment-scale analyses may not be easily applied [*Beven*, 3; *Bloschl and Sivapalan*, 4; *Famiglietti and Wood*, 19–20; *Gupta et al*., 1986; *Dooge*, 13]. In basins of such immense size, large-scale land-atmosphere interactions may play a critical role in the retention of water and changes in storage during the wet season [*Trenberth et al*., 48; *Makarieva and Gorshkov*, 28; *Eltahir and Bras*, 18].

[9] NASA’s Gravity Recovery and Climate Experiment (GRACE) mission [*Tapley et al*., 46] now provides the opportunity to observe the dynamics of water storage for large river basins. With monthly global coverage, GRACE data are well suited to contribute to better understanding of hydrology at the larger temporal and spatial scales that are important for climate studies [*Lettenmaier and Famiglietti*, 26]. For the current research, we focused on developing new methods to explore the information contained in the GRACE data at its intrinsic large-basin scale, in hopes of improving our conceptual understanding of the global water cycle.

[10] In this study, we begin with the hypothesis that precipitation alone does not drive large-scale storage variability, but that the influence of precipitation forcing is affected significantly by the land surface with which the water interacts. We test this with an analysis of the impact of land-surface variables on large-basin water balance, represented by the propagation of variability from Global Precipitation Climatology Project (GPCP) precipitation time series [*Adler et al*., 1] to GRACE water storage time series. We then use land-cover, soil properties, and terrain information to draw broad conclusions about the large-scale land-surface impact on water balance.

**Figure 1:**
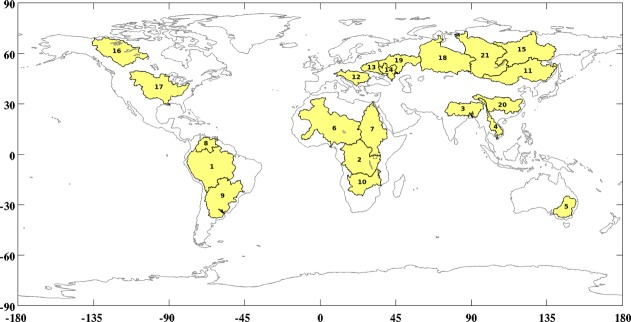
Map of study basins. (1) Amazon, (2) Congo, (3) Ganges and Brahmaputra, (4) Mekong, (5) Murray, (6) Niger, (7) Nile, (8) Orinoco, (9) Parana, (10) Zambezi, (11) Amur, (12) Danube, (13) Dnieper, (14) Don, (15) Lena, (16) Mackenzie, (17) Mississippi, (18) Ob, (19) Volga, (20) Yangtze, and (21) Yenesei.

[11] First, basin-averaged time series are estimated for 21 of the world’s largest catchments (Figure 1) and analyzed for the propagation of variability at two timescales, based on frequency-domain transfer functions. These transfer functions are then parameterized to understand the influence of basin-mean temperature (from the National Centers for Environmental Prediction (NCEP) [*Kalnay et al*., 24], percent forest cover within a basin derived from Moderate Resolution Imaging Spectroradiometer (MODIS) satellite observations [*DeFries et al*., 12], soil water-holding capacity (WHC) [*Dunne and Willmott*, 15], and basin terrain characteristics. Finally, we validate generalized parameterizations by modeling storage time series and comparing with observations from GRACE.

[12] In addition to contributing to understanding of large-scale hydrology, this study enables the prediction of future water storage variations given climate model predictions of precipitation [*Taylor et al*., 47]. There is also the potential for a multiyear gap between the failure of the current GRACE mission and the launch of its successor. The methods described here could be used to estimate water storage variations within this intermission time period. Although the current GRACE record is short and causes difficulty in the application of time series analysis methods, it is still critically important to offer a statistical model of the data with the best methodology available, given the critical need to understand water storage variations in the future.

## 2. Data and Methods

### 2.1 Methods’ Overview

[13] In order to investigate storage behavior across basins, we use an empirical approach to analyze and model the GRACE observations. Rather than solving equation (1) for d*S*/d*t*, we instead seek an estimate of storage response *S*(*t*) that is normalized for precipitation forcing. We achieve this objective through the following steps: First, a frequency-domain analysis is performed, including a dynamical systems model (single input/output cross-spectral analysis) for basin-averaged precipitation-storage transfer functions. Second, we select a small list of probable parameters to create a functional relationship between measured storage response and lumped basin variables. Third, we apply modeled transfer functions to observed precipitation spectra and return to the time domain with a prediction of storage anomaly.

### 2.2. GRACE

[14] Because of the inherent spatial limitations of the GRACE data [*Wahr et al*., 52], we use scaled basin-averaged time series [*Swenson and Wahr*, 41] for basins with a drainage area greater than 200,000 km^2^ and a drainage volume of greater than 40 km^3^/yr. Because of two significant and distinct causes of error in the GRACE data, GRACE basin time series must be corrected for loss of signal due to measurement error (based on the GRACE footprint) and for “leakage” error (the contamination of a signal by adjacent stronger signal). Using uncorrected (“unscaled”) estimates of basin storage will lead to an erroneous representation of GRACE-derived water balance. The error correction is performed by creating a linear scaling operator for the GRACE basin-averaged data [*Swenson and Wahr*, 42; *Wahr et al*., 51]. In summary, synthetic basin variability is converted into a global spherical harmonic solution to degree and order 60 and smoothed spatially with a Gaussian filter at 300 km radius. The processed output is then compared to the raw input to create a scaling parameter that will be applied to similarly processed GRACE observations. Results have been validated with in situ observations in several studies [*Famiglietti et al*., 21; *Swenson et al*., 43; *Yeh et al*., 53].

[15] Following the processing for basin averages, the GRACE data are best suited for application in the following large basins (Table1 and Figure 1): Amazon, Amur Congo, Danube, Dniepr, Don, Ganges and Brahmaputra, Lena, Mackenzie, Mississippi, Murray, Niger, Nile, Ob, Orinoco, Parana, Volga, Yangtze, Yenisei, Zambezi, and Mekong. The Murray is included even though it has a drainage volume of only 9 km^3^/yr. The effects of this are discussed in the analysis.

**Table 1:** A List of the Study Basins, With Basin Drainage Area and Drainage Volumes Estimated at River Mouth [*Dai and Trenberth*, 2001], Basin-Mean Temperature, Forest Cover, and Soil WHC^a^

Basin	Drainage Volume (km^3^/yr)	Drainage Area (10^3^ km^2^)	Basin-Mean Temperature (°C)	Basin-Mean Forest cover (%)	Basin-Mean Soil WHC (cm)	GRACE Observation Error (cm)	Annual Variability Admittance	Interannual Variability Admittance	Model *r*^2^	Model *E_f_*
Amazon	6642	5854	19.6	79.5	10.0	1.1	0.70 ± 0.02	0.15 ± 0.18	0.92	0.84
Congo	1308	3699	19.5	62.1	9.2	1.5	0.52 ± 0.06	0.36 ± 0.04	0.84	0.70
Gang/Br	404 + 628	956 + 583	16.4	25.3	8.3	1.9	0.45 ± 0.02	0.34 ± 0.07	0.80	0.64
Mekong	525	774	18.7	40.9	9.7	2.5	0.50 ± 0.03	0.31 ± 0.05	0.89	0.79
Murray	9	1032	17.1	19.0	7.9	1.7	0.03 ± 0.13	0.32 ± 0.05	0.71	0.61
Niger	193	2240	25.6	8.0	7.0	1.6	0.47 ± 0.02	0.45 ± 0.12	0.89	0.79
Nile	40	3826	23.6	7.7	6.7	1.5	0.36 ± 0.06	0.39 ± 0.06	0.77	0.58
Orinoco	1129	1039	22.7	63.0	10.8	1.8	0.65 ± 0.02	0.01 ± 0.19	0.74	0.54
Parana	568	2661	19.5	31.4	8.4	1.9	0.26 ± 0.05	0.43 ± 0.06	0.83	0.68
Zambezi	117	1989	20.4	24.5	9.1	2.2	0.44 ± 0.03	0.40 ± 0.10	0.88	0.75
Amur	354	2903	1.4	35.2	6.0	1.1		0.07 ± 0.07	0.57	0.28
Danube	202	788	8.1	32.3	15.2	2.0		0.32 ± 0.17	0.39	0.11
Dnieper	47	509	7.2	20.6	14.8	2.6		0.62 ± 0.16	0.82	0.66
Don	45	423	7.6	7.5	13.3	2.8		0.25 ± 0.09	0.80	0.62
Lena	531	2418	−6.2	70.3	2.9	1.1		0.07 ± 0.19	0.18	0.02
Mackenzie	290	1713	−2.3	42.3	12.6	1.3		0.17 ± 0.10	0.31	0.09
Mississippi	610	3203	10.6	28.4	17.6	1.1		0.31 ± 0.12	0.65	0.42
Ob	412	2570	0.7	41.8	11.5	1.1		0.28 ± 0.10	0.37	−0.08
Volga	254	1380	4.0	40.5	12.4	1.8		0.30 ± 0.07	0.70	0.48
Yangtze	944	1794	10.4	30.7	7.4	1.3		0.11 ± 0.21	0.51	0.26
Yenisei	599	2582	−3.2	61.6	5.0	1.2		0.53 ± 0.46	0.14	−0.08

a Also, the GRACE observational error, and annual period and interannual period admittance with 95% confidence intervals from transfer function solutions. Finally, the squared correlation coefficient and Nash-Sutcliffe efficiency between predicted and observed storage.

[16] The inherent temporal limitations of the GRACE data (one robust solution per month) effectively limit the usable resolution of the other data sets to monthly as well. Note that the GRACE data are not sampled once monthly in each location, but that several samples (three to four) approximate to the area of interest are taken within the month. These are combined to create a single global solution for GRACE water storage anomaly at a monthly interval, which we assume for this study to represent a monthly average.

### 2.3. Precipitation

**Figure 2:**
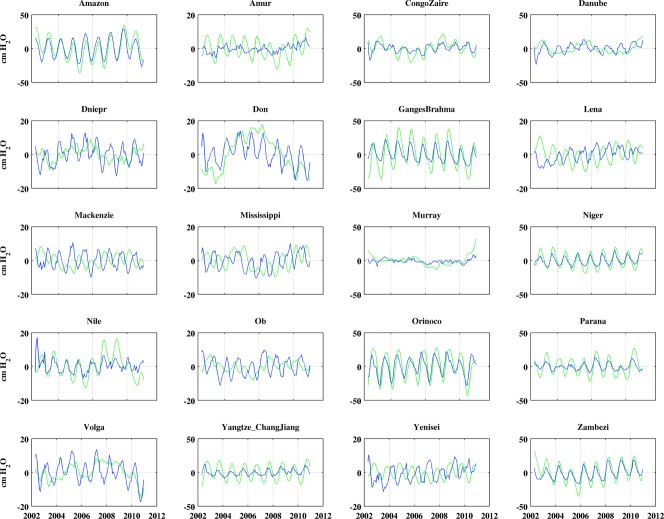
Basin-mean time series of storage anomaly from GRACE (blue) and cumulative precipitation anomaly from GPCP (green).

[17] Global precipitation observations are taken from the GPCP [*Adler et al*., 1]. Global grids of 2.5° monthly mean precipitation were downloaded from http://www.esrl.noaa.gov, and masks for each of the study basins were applied to estimate basin-averaged time series. The basin-averaged precipitation time series (mm/d) were converted to a cumulative precipitation anomaly by first removing the mean and then integrating with respect to time. This creates an estimate of the variability of the total precipitation input (cm) during the GRACE record and results in units of height anomaly that match those of storage (Figure 2).

### 2.4 Temperature Data

**Figure 3:**
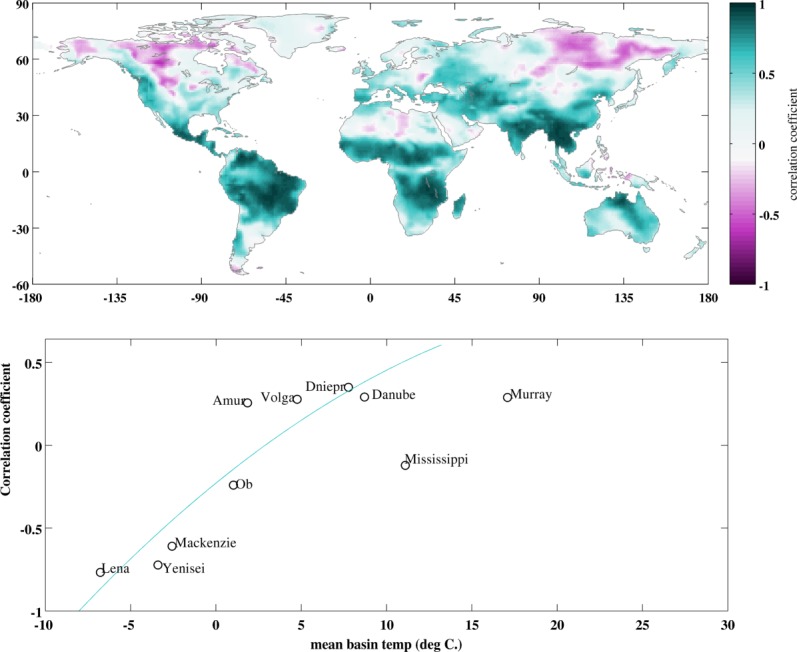
Time series correlation coefficients between cumulative precipitation and storage anomaly: (top) at 1° resolution globally and (bottom) for selected study basins as a function of basin temperature.

[18] Global gridded 1° temperature data come from the NCEP reanalysis [*Kalnay et al*., 24]. These data were used to calculate basin-averaged temperature to compare against precipitation-storage behavior. This comparison is shown as a map (Figure 3, top) and as a plot (Figure 3, bottom).

### 2.5. Land-Surface Variables

[19] We investigated several candidate land-surface variables in order to explore which had the greatest influence on controlling terrestrial water storage at the large scales observed by GRACE. In modern land-surface models, water balance losses are controlled by a small list of parameters: soil matric potential, soil porosity, and soil depth; land-cover type; and topography. We represent those parameters here in a gross fashion: one parameter for soil (*soil w.h.c*.), one parameter for vegetation (*% forest cover*), and one for topography (*surface slope*). We also add *basin size*, which is relevant in a realistic characterization of basin behavior that includes surface water storage. Each is described later.

#### 2.5.1. Percent Forest Cover

**Figure 4:**
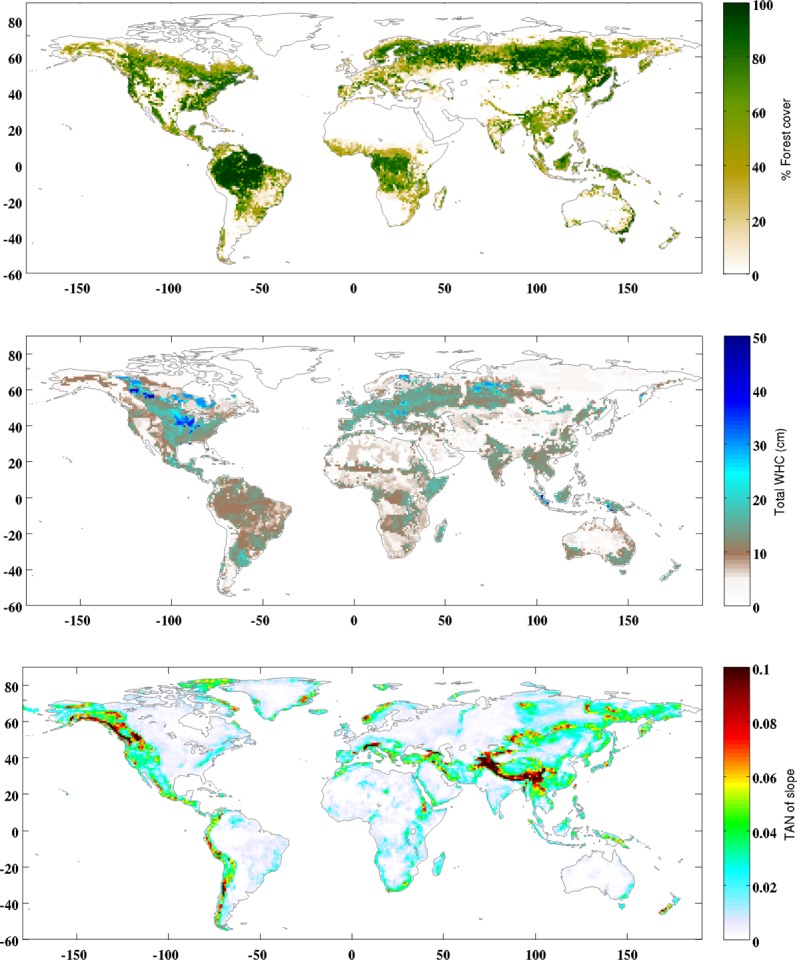
Maps of tested parameter variables: (top) global percent forest cover from MODIS, (middle) global plant-available soil WHC (in centimeters) from *Dunne and Wilmott* [15], and (bottom) global terrain slope (tangent of slope) from Hydro1k. All are estimated at 1° resolution.

[20] Percent forest-cover data were downloaded from University of Maryland Global Land Cover Facility [*DeFries et al*., 12] at 1° resolution with global coverage and are shown in Figure 4a. Unlike land-cover type classification, forest cover is a continuous variable and can be averaged within a basin to achieve a basin-mean estimate. This approach assumes that a linear average of subbasin land cover will approximate the total basin land-cover effect on storage response.

#### 2.5.2. Soil Water-Holding Capacity

[21] Soil WHC [*Dunne and Willmott*, 15] data were downloaded from the NASA Global Change Master Directory at http://gcmd.nasa.gov/ and are shown in Figure 4b. These data are calculated empirically based on plant-extractable water capacity of soil—the maximum amount of water that can be extracted from the soil to fulfill evapotranspiration demands—and serve as a proxy for relative soil water storage capacity across the study basins.

#### 2.5.3. Mean Terrain Slope

[22] Global terrain slope data at 1° resolution were downloaded from the ISLSCP II data set website (http://daac.ornl.gov/). These data are derived from the Hydro1k digital elevation model [*Verdin*, 50]. Hydro1k has a native spatial resolution of 1 km, the highest resolution database with global coverage of standard elevation-based derivatives. Figure 4c shows the global distribution of derived terrain slopes from which we constructed basin averages for comparison with GRACE data.

#### 2.5.4. Basin Area and Drainage Volume

[23] Basin area and drainage volume [*Dai and Trenberth*, 11] were also investigated as potential controls on relative storage response and are listed in Table1.

### 2.6. Fourier Analysis

#### 2.6.1. Basin-Averaged Water Storage and Precipitation Spectra

[24] The goal of spectral analysis is to describe the distribution (in frequency) of the variability contained in a signal during a finite observation period. The basin-averaged GRACE time series were transformed into frequency domain to investigate the dominant frequencies in the GRACE signal. Because the GRACE record is short (late 2002–present), and because we are limited to a monthly sampling frequency, our resulting data series contain a low number of samples for standard frequency-domain analysis. Using a parametric spectral method instead of a nonparametric method, we can resolve undersampled frequencies with a single complete instance in the time series and sufficient energy, to reduce the impact of noise in a short time series that could cloud results in traditional spectral approaches. We apply the Yule-Walker autoregressive method of spectral estimation [*Emery and Thomson*, 16] across the entire time series to detect significant variability down to 1 cycle/5 yr. This parametric method is better for short time series than traditional power spectra, but interpretation of results requires some a priori knowledge of the dominant spectral periods.

**Figure 5:**
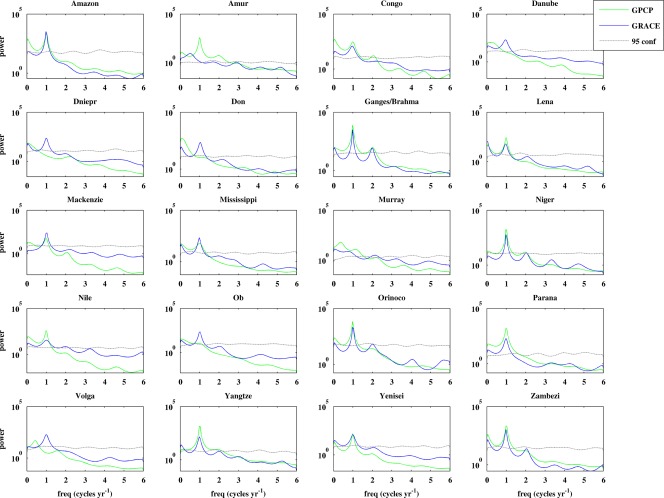
Basin-averaged spectra for storage anomaly for GRACE (blue) and cumulative precipitation anomaly from GPCP (green). The dashed line is the spectra for a white-noise time series with twice the standard deviation of the observations.

[25] In order to estimate confidence in the results, we also calculate the Yule-Walker spectrum for a white-noise time series that possesses twice the standard deviation (95% confidence) of the basin-averaged GRACE storage. The scaled confidence spectra are shown in Figure 5 as a dashed line. Based on these results, the statistically significant spectral energy was grouped into two frequency ranges for the modeled storage response in section 2.6.4: annual (1 year period) and interannual (2.5–5 year period).

#### 2.6.2. Basin-Averaged Transfer Functions

**Figure 6:**
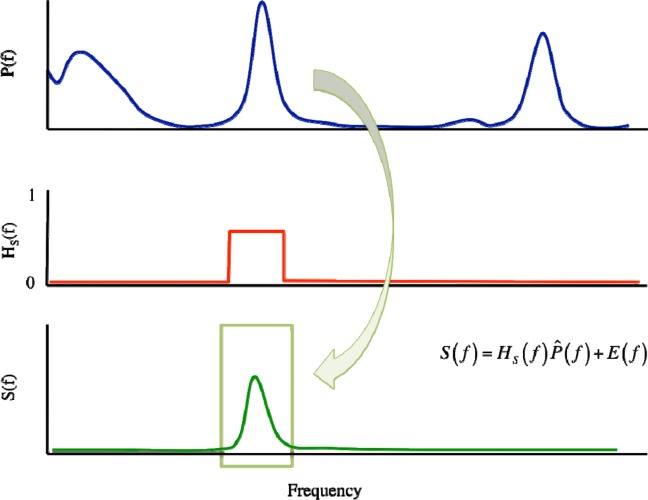
Theoretical transfer function example, showing the propagation of variance from precipitation to storage in a specific frequency range.

[26] Here we define a transfer function as a unit-response function from precipitation input to storage output, which, over a range of inputs, describes the response of the land surface to precipitation variability. It is limited in that it only quantifies periodic signals, not trends or biases. It is also limited by considerations of ensemble size and averaging window width, which vary based on available record length. Transfer functions for a precipitation input and a storage output, assuming wide-sense stationary and stochastic processes, were calculated using


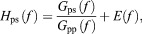
(2)

where *G*_PP_ represents the two-sided autospectra of basin-averaged precipitation anomaly, *G*_PS_ represents the two-sided cross-spectra of basin-averaged precipitation anomaly and storage anomaly, and *E* is an error term for unexplained variance. Two-sided spectra are defined such that the frequency range of integration ranges from negative to positive infinity. The result occupies the whole Nyquist interval [to 1/d*t*] with a symmetrical spectrum in the second half, as opposed to the single-sided spectrum that goes to the Nyquist frequency [1/(2d*t*)]. Figure 6 offers a conceptual depiction of the transfer function operation. Transfer functions were calculated here using the Welch periodogram method [*Emery and Thompson*, 16] for cross-spectra and autospectra. This allows the calculation of confidence intervals based on record length.

[27] A two-sided transfer function yields phase data in the imaginary component of the solution. To simplify transfer function interpretation, we assume that there is no time lag possible between precipitation forcing and storage response in snow-free basins. If rain falls in a basin, the basin storage changes simultaneously, like water in a bucket responding to filling by a hose. In order to apply this rule, we have allowed for only zero-time-lag propagation of precipitation variability to storage variability, by applying the imaginary portion of the transfer function correctively. Because the transfer function depends on the cross-spectra in its calculation, it contains the phase information for the two time series. We can discard the orthogonal, imaginary portion of the transfer function from the total magnitude, to remove the nonzero-time-lag portion of the signal. We are then left with only the instantaneous (monthly) basin storage response per unit precipitation forcing at each discrete frequency interval.

[28] For this analysis, we consider the higher-frequency signal in the transfer functions—beyond a frequency of 2 cycles/yr (6 month periodicity)—as noise. This is in order to simplify our results to the major periodic frequency ranges that appear in the GRACE data. Since the transfer functions represent an “admittance” of signal between input and output, even a small signal due to processing noise can appear in the result. Hence, we discount weaker frequencies as insignificant and concentrate on the significant discrete frequencies from the spectral results: the annual period and a combination of interannual (2.5–5 year) periods. We then regard the transfer function estimates with their corresponding confidence ranges.

#### 2.6.3. Parameterized Transfer Functions

[29] The correlations between transfer function admittance (the propagated variability from precipitation to storage) and a range of values in certain basin-averaged parameters allow the formulation of a least squares solution. We applied a logarithmic fit for transfer function admittance as a function of the correlated land-surface variables. This allowed us to reconstruct a synthesized transfer function based on those parameters and apply this modeled transfer function to a precipitation time series as described in section 2.6.4.

#### 2.6.4. Reconstructed Storage Time Series

[30] To empirically reconstruct a storage time series, we estimate the discretized transfer function at the annual and interannual (2–5 year) ranges based on the empirical model equations from equation (2). We then convert a precipitation forcing time series into the frequency domain using a fast-Fourier transform. The smoothed transfer function is multiplied by the precipitation spectrum to create an output storage spectrum. The inverse fast-Fourier transform is applied to the results to recreate a time domain series of basin-averaged storage. Transfer function confidence intervals are carried through the process.

## 3. Results

### 3.1. Fourier Analysis

[31] Results of the spectral estimates of the GRACE and GPCP data are shown in Figure 5. The first noticeable congruency among all of the spectral series is a general lack of high-frequency variability with significant peaks at the annual and interannual periods. All of the study basins have significant energy at the interannual period (less than 1 cycle/yr) for both time series, except for the Amazon, Mackenzie, Niger, Orinoco, and Volga.

[32] The fact that the highest peaks occur at the annual period in almost all of GRACE storage time series (the blue lines in Figure 5)—even those that do not have significant spectral energy at the annual period in precipitation—deserves discussion. While the single greatest influence on shaping storage spectra is precipitation, we see in Figure 3 (bottom) that temperature has the ability to decorrelate the precipitation and storage time series and also their spectra. Several spectra with no significant energy at the annual period in precipitation have significant energy in the annual period for storage (Ob, Danube, Dnieper, Don, Volga). This is caused by the action of temperature to accumulate snowfall during the winter and discharge it during the spring, integrating the faster timescale precipitation variability into the 1/yr frequency.

[33] Because of the dominant ability of freezing temperatures to modulate annual storage, in the rest of the analysis, we chose to separate the basins into two categories: “warm” basins (those with a mean temperature greater than 15°C), and “cold” basins (those with a mean temperature less than 15°C). The 10 warm basins are those clustered toward the right of Figure 3 (top), showing a good correlation with storage. The map in Figure 3 (bottom) gives spatial detail on the relationship between temperature and storage, which shows even negative correlations for precipitation and storage at higher latitudes.

**Figure 7:**
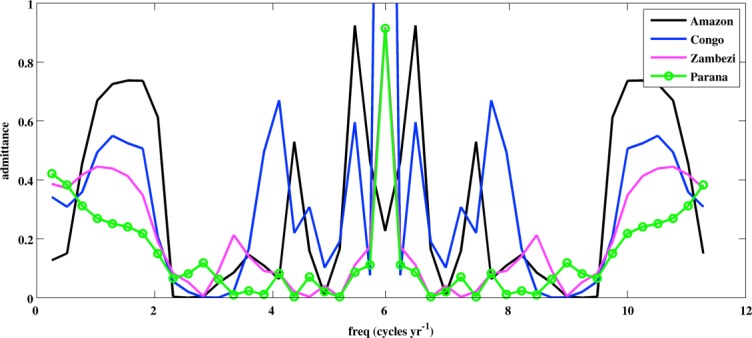
Basin-mean transfer function admittance for precipitation input to storage output for four basins, plotted as a function of frequency (cycles/yr).

[34] Figure 7 shows transfer function solutions for four warm basins (Amazon, Congo, Parana, and Ganges-Brahmaputra), as a function of frequency. The Amazon has the transfer function with the most admittance at the annual period, and the Parana with the least. However, there is a critical threshold moving into the low-frequency range beyond which the Parana becomes the most active transfer function and the Amazon the least. This highlights an important result: not only do basins have a different response to precipitation based on their land-cover and soil characteristics, but these responses also vary, or show some nonlinearity, across timescales.

### 3.2. Uncertainty and Assumptions

[35] The error estimates in GRACE data are constant in time for a region, as they exist primarily as a function of the GRACE footprint and limit resolution defined by the orbital configuration of the satellites. They are therefore assumed not to affect the storage response in different frequency ranges differently (i.e., leakage and smoothing errors are assumed constant in time for a region). In other words, while significant error exists in the GRACE data, it is assumed to have negligible frequency content and not to propagate into the frequency domain. The time-constant observational errors included in Table1 should be used as a proxy for observation error in the predicted time series of GRACE terrestrial water storage for each of the study basins.

**Figure 8:**
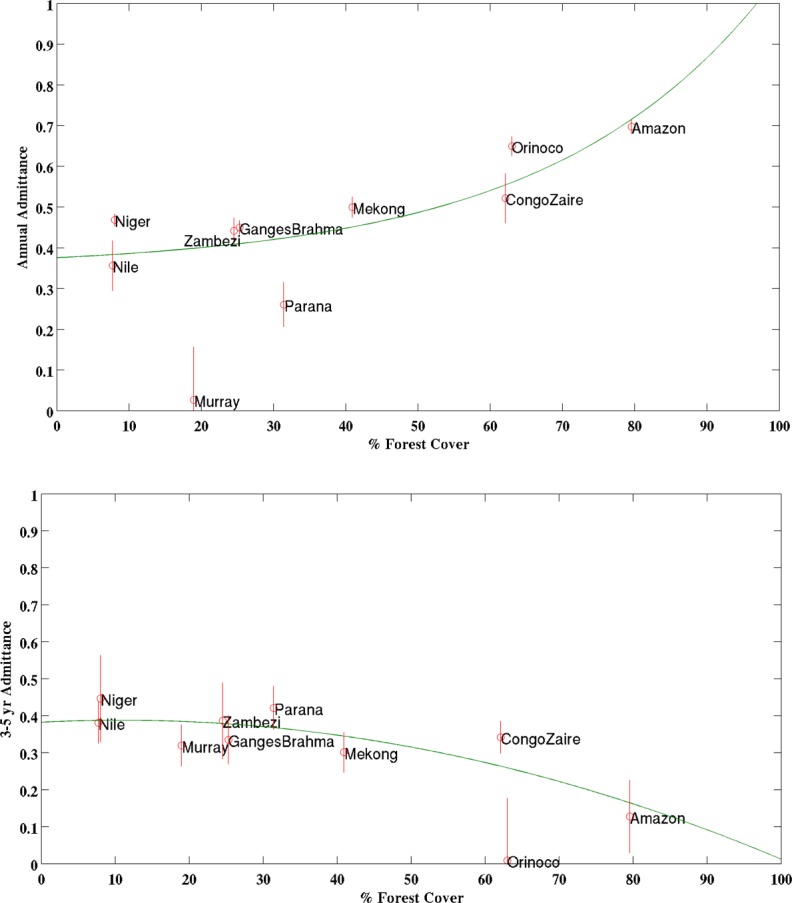
Basin-mean storage response to precipitation forcing (transfer function admittance) as a function of percent forest cover. Shown for the annual and low-frequency timescales. The best fit model function is also plotted.

**Figure 9:**
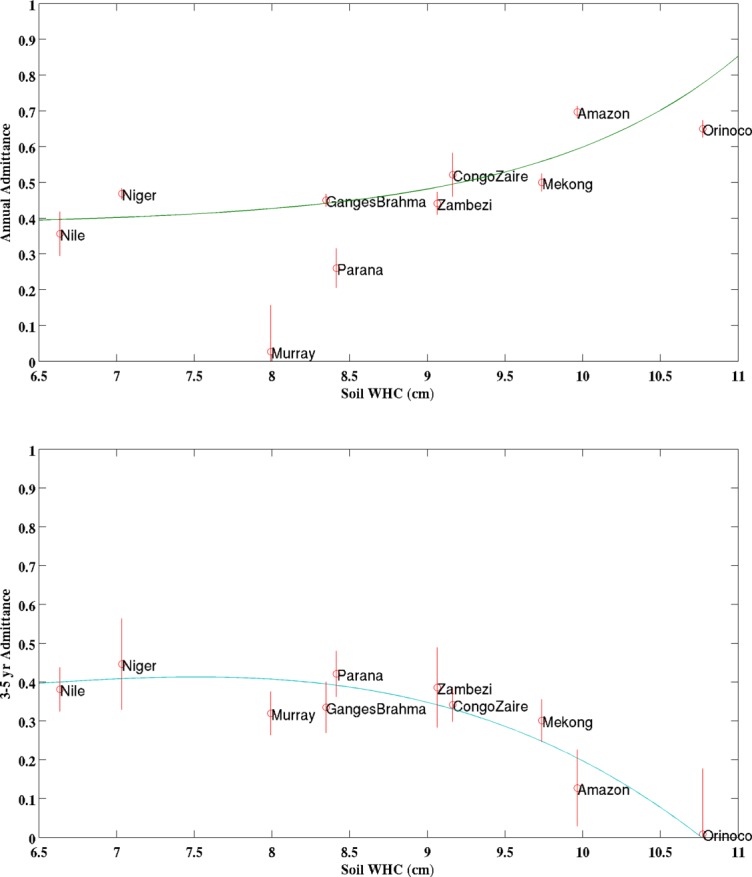
Storage response to precipitation (transfer function admittance) as a function of basin-mean soil WHC. Shown for the annual and low-period timescales. Best fit model is also plotted.

**Figure 10:**
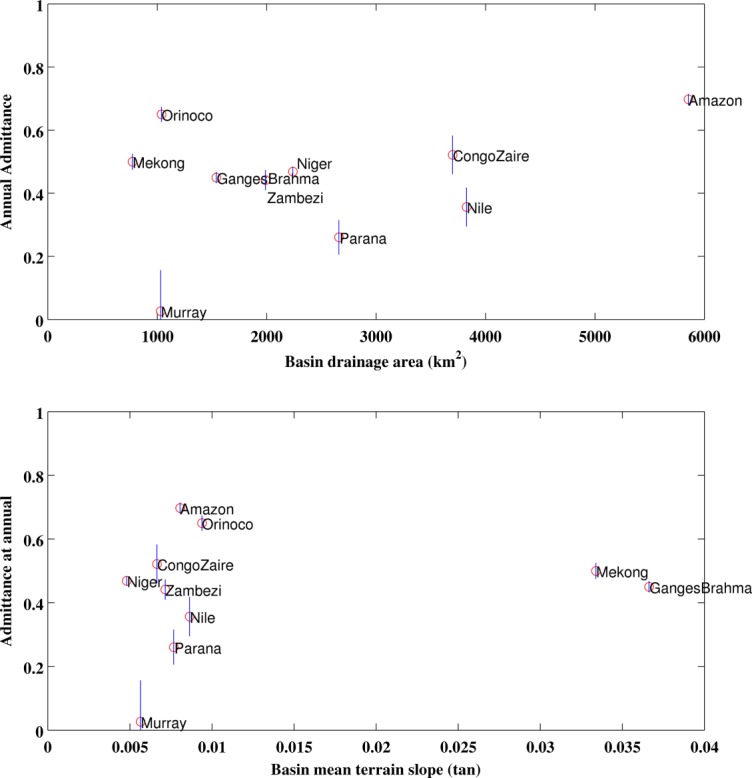
Annual period transfer function admittance as a function of (top) basin drainage area and (bottom) basin-mean terrain slope.

[36] Frequencies with statistically significant spectral energy in the GRACE and GPCP time series were included in the transfer function results. The confidence estimates of the transfer function solutions were calculated assuming a *χ*^2^ distribution of random error for the quality of the spectral solution relative to the length of the observational record and corrected for windowing. The 95% confidence intervals for the transfer functions are plotted as error bars in Figures 8, 9, 10.

### 3.3. Suggested Controls on Rainfall-Storage Relationship

[37] Among the variables tested, temperature appears as the single most important characteristic controlling storage across basins in our study, and the correlation between precipitation and storage decays quickly as basin-mean temperature decreases. In summation, zero-lag transfer function admittance from precipitation to storage varied widely across cold basins. Storage in these basins was highly anticorrelated with temperature time series at 120 day lag times (temperature leading storage), indicating a strong seasonal response of storage to temperature forcing, and as mentioned earlier, often uncorrelated or anticorrelated with precipitation seasonality.

[38] In Figure8, we see the effects of percent forest cover on transfer function admittance for two different timescales, for each of the warm study basins. Land-cover density has the effect of preserving relatively more precipitation variability in storage at the annual period and less precipitation variability in storage at longer timescales. For the cold basins, there was little to no admittance at the annual period due to the uncorrelated time series, and the zero-time-lag rule applied here. However, for the cold basins at the interannual timescale, the effect of vegetation density is generally the same as for the warm basins.

[39] The plots of transfer function admittance versus available soil water are shown in Figure9. The effect of soil WHC is very similar to that of forest cover. As soil WHC increases, the admittance of variance from precipitation to storage at the annual period also increases. For interannual timescales, this effect reverses, and basins with more WHC show a reduced storage response to precipitation forcing.

[40] Basin storage response relative to basin size and topography is shown in 10 for annual period admittance only. Basin slope and basin drainage area had no significant correlation with transfer function admittance at either the annual or interannual period.

### 3.4. Modeling Storage Response to Precipitation Forcing

[41] We use our generalized transfer functions at two frequency ranges to create new time series of terrestrial water storage for warm and cold regions, based on land-cover type, forest cover, soil WHC, and given a precipitation input time series. For extended time periods, these predicted storage time series neglect the effects of any significant feedbacks on storage from ecosystem change and assume a stable vegetation range for an ecosystem during the GRACE record.

[42] We first fit empirical functions with an assumed logarithmic shape to the transfer function results across two correlated variables: basin-mean percent forest cover and basin-mean available soil depth. The model is applied to the warm basins only, since warm basins are more clearly correlated with land-surface parameters for their storage response across all frequency ranges, except for the Murray (due to the anomalously low correlation with precipitation at the annual frequency). Once the parameters were estimated, the model was applied to all basins. The best fit functions are shown in Figures 8 and 9. We use a logarithmic function of the form:


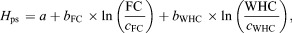
(3)

where *H*_PS_ is the admittance of variability from precipitation to storage, FC and WHC are the values of the land-surface parameter (forest cover and WHC), and *a*, *b*, and *c* are model parameters. This model was applied to the annual and low-frequency storage response.

**Figure 11:**
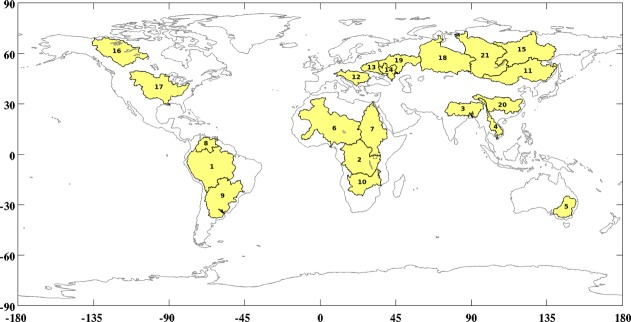
Basin-modeled storage anomaly time series for warm basins (red) and 95% confidence (gray shaded), based on cumulative precipitation anomaly (blue), compared with observed storage anomaly from GRACE (black dashed). Storage is in centimeters equivalent of water over the basin.

[43] The model predicted storage time series are shown in Figure 11 for warm basins and 12 for cold basins. For cold basins, only the interannual storage variability was modeled, since only the low-period transfer functions were correlated with land-surface parameters. The model results (red) with 95% confidence interval (gray shaded) are compared to the GRACE observations (black). Validation metrics were calculated against a GRACE signal smoothed for coherence with the designed model (i.e., considering only the modeled frequencies in both the synthetic and observed GRACE data). The correlation coefficients (*r*^2^) and model efficiency (*E_f_*) [*Nash and Sutcliffe*, 32] between modeled and observed storage anomaly are listed in Table1.

**Figure 12:**
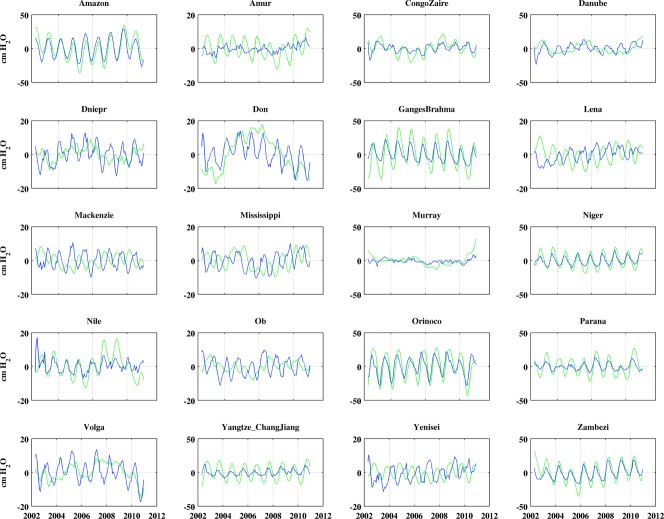
Basin-modeled storage anomaly time series for cold basins (red) and 95% confidence (gray shaded), based on cumulative precipitation anomaly (blue), compared with observed storage anomaly from GRACE (black dashed). Storage is in centimeters equivalent of water over the basin.

[44] In warm basins, the generalized model was able to correctly reproduce the magnitude of the annual variability in GRACE observations and a portion of the interannual period variability. For warm basins, model results were generally well correlated (0.71 ≤ *r*^2^ ≤ 0.92) with observations and accurate (0. 54 ≤ *E_f_* ≤ 0.84). For cold basins, in comparison to the GRACE interannual signal, model results were moderately correlated (0.14 ≤ *r*^2^ ≤ 0.82) and had mixed accuracy (−0.08 ≤ *E_f_* ≤ 0.66).

## 4. Summary and Discussion

[45] When viewed across the spectrum of global river basins, the rainfall-storage relationship is quite complex. While two areas may receive similar amounts of precipitation, there can be differences in the resulting storage response due to temperature and the characteristics of the land surface. The basins used in this study are extremely large and give a general representation of land-cover types, across climates, globally.

[46] To summarize, we list the order of importance of controls on basin terrestrial water storage variability at mega-basin scales as follows: (1) temperature: in regions colder than 15°C, temperature-driven storage acts to generally decouple storage variability from precipitation forcing because of snow accumulation. For periods longer than annual, temperature becomes a less significant factor in storage response. (2) Land cover: Denser forest cover is correlated with less long-period storage variability and a more consistent annual dynamic storage range. (3) Soil: soil depth and porosity, combined here as soil WHC, are correlated with less long-period storage and a more consistent annual dynamic storage range. (4) Topography: terrain slope and basin area were found to have an insignificant correlation with storage behavior at the large-basin scale.

[47] At the lowest order, temperature acts in a grossly binary fashion on basin storage: either to accelerate water loss through ET or to prevent water loss through snow accumulation. Our results show that in colder basins, such as the Mackenzie, temperature causes a decorrelation or even an anticorrelation between precipitation and storage time series. In the winter, higher-frequency precipitation variability is integrated and redistributed temporally into a cumulative seasonal signal, and gravity-based storage observations reflect this well-known effect. With the onset of springtime temperature increases, runoff and evaporative losses resume and drive decreasing storage, sometimes concurrent with (but often outpacing) rainy season precipitation increases. This effect acts at the annual period, while at interannual periods storage variability is better correlated with interannual precipitation variability. This effect makes it difficult to decompose GRACE storage spectra into its component pieces and difficult to model storage time series in cold regions from precipitation input. An interesting continuation of our study would involve representing temperature influence on storage empirically, or alternatively, using storage observations to tune ET losses in a land-surface model.

[48] Over a typical annual cycle, more water remains in storage per unit of precipitation with more forest cover and with more soil capacity. We find this effect more significant than that of basin area on storage response. These results suggest, for example, that a relatively small but well-forested basin like the Orinoco can maintain water in storage (per unit precipitation) during a typical rainy season that a larger, less forested basin such as the Zambezi or Parana cannot.

[49] The discrepancy between interannual behavior and annual behavior in the transfer functions suggests a difference in basin storage capacities across water-limited and nonwater-limited ecosystems. An interannual storage signal is not truly independent from an annual storage signal and in reality, is occuring with the annual storage signal for each monthly data point. For example, the lack of transfer of an interannual signal from precipitation to storage may indicate a full storage capacity in a given month. Despite an extreme in precipitation, some finite range of storage variability has been met, and now further storage gains are limited by the flooding of water out of the basin or by increased evaporation. In the tropics, interannual precipitation events (like those linked to El Niño–Southern Oscillation) may drive interannual increases in runoff or evapotranspiration, resulting in little interannual change in storage. A relatively larger interannual storage signal indicates the opposite mechanism: a basin that is often water-limited.

[50] Certainly, it is well known that basin slope has an impact on river runoff and therefore on storage. For instance, in the Mekong basin [*McGuire et al*., 30], as well as in voluminous catchment-scale studies, terrain slopes are important in runoff modeling. The absence of influence in these results could be due either to the very large scale of the basins studied (i.e., that terrain variability in the mega-basins is simply not great enough to impact large-scale storage variations at the timescales investigated here); to the relatively low resolution of the data sets required to conduct a global study such as this one (so that some important terrain attributes were not well resolved); or to the fact that a broader sample of terrain attributes was not included in the research. It is also likely that basin slope has the most immediate effect on runoff generation, and in steep basins, precipitation extremes are matched by runoff extremes, resulting in little storage effect.

[51] There is a critical basin size and discharge volume range in which this model of storage variability applies. For example, the transfer function of the Murray basin is a particularly poor fit at the annual period resulting in a large confidence interval on admittance. This is likely due to the low flow volume of the Murray (Table1) and the high degree of water management within the basin (Australia diverts river flow for agriculture and was in a severe drought from 1995 to 2009) [*Ummenhofer et al*., 49]. As such, the storage signal does not possess much of the natural forcing variability from the precipitation input. It is interesting to note though that at the interannual period, the observed Murray basin response falls in line accurately with the parameterized transfer function fit. One has to consider that while precipitation seasonality can be expected and managed, the prediction of interannual precipitation variability is more difficult, and reservoir storage is limited by capacity in time. For these reasons, management may have its strongest impact on storage variability at the seasonal timescale.

[52] Our results showing the importance of land-atmosphere interaction in large basins are supported by previous work. *Eltahir and Bras* [17] had shown precipitation recycling estimates of 25%–35% in the Amazon, and *Makarieva and Gorshkov* [28] and *Makarieva et al*. [29] have shown that for large basins, precipitation recycling is a necessary mechanism to bring water deeper than 600 km into a basin. It is likely that large-scale recycling is a mechanism which retains more water in storage in relatively wet regions and that this effect accounts for the larger annual period response in well-forested basins. This would signify that large-scale basin water dynamics are entirely distinct from field-scale or even regional-catchment-scale dynamics.

[53] While estimation of basin residence times based on these data sets was desired, it is not possible to do so quantitatively due to the fact that GRACE observes only anomalies of water storage, never an absolute amount, and the application of even a simple linear reservoir model requires more information. It is possible however to come to qualitative inferences about the relative residence tendencies for water storage across the study basins. The dynamics of the land-atmosphere link and the ability of large, well-forested basins to retain water in storage for long periods through recycling are important in large-scale hydrology, and suggest longer residence times in those basins. While a catchment-scale study may not consider atmospheric dynamics, land-atmosphere interactions, or precipitation recycling, for global-scale basins, these processes are probably of critical importance.

[54] Based on our results, we can now predict a monthly storage anomaly time series using GPCP precipitation time series and two land-surface variables (*percent forest cover* and *soil water holding capacity*) in the world’s mega-basins. Storage time series for large basins are uncommon and of use to water managers for understanding total water availability, and to large-scale hydrologic or land-surface modelers as a state variable for calibration and validation. While the simple model we have presented here does not account for nonlinear transitions in land-cover type or the evolution of terrestrial ecosystems, it offers the first means for predicting typical storage variability time series within the limits of a stable ecosystem.

## References

[b1] Adler RF, Huffman GJ, Chang A (2003). The Version-2 Global Precipitation Climatology Project (GPCP) monthly precipitation analysis (1979–present). J. Hydrometeorol.

[b2] Atkinson SE, Woods RA, Sivapalan M (2002). Climate and landscape controls on water balance model complexity over changing timescales. Water Resour. Res.

[b3] Beven K, Kalma JD, Sivapalan M (1995). Linking parameters across scales: Subgrid parameterizations and scale dependent hydrological models. Scale Issues in Hydrological Modeling.

[b4] Bloschl G, Sivapalan M, Kalma JD, Sivapalan M (1995). Scale issues in hydrological modeling: A review. Scale Issues in Hydrological Modeling.

[b5] Bonan GB (2008). Forests and climate change: Forcings, feedbacks, and the climate benefit of forests. Science.

[b6] Bonan GB, Oleson KW, Vertenstein M, Levis S, Zeng X, Dai Y, Dickinson RE, Yang Z-L (2002). The land surface climatology of the community land model coupled to the NCAR community climate model. J. Clim.

[b7] Budyko MI (1974). Climate and Life.

[b8] Chen JL, Wilson CR, Tapley BD, Yang ZL, Niu GY (2009). 2005 drought event in the Amazon River basin as measured by GRACE and estimated by climate models. J. Geophys. Res.

[b9] Cherkauer KA, Lettenmaier DP (1999). Hydrologic effects of frozen soils in the upper Mississippi River basin. J. Geophys. Res.

[b10] Crowley JW, Mitrovica JX, Bailey RC, Tamisiea ME, Davis JL (2006). Land water storage within the Congo Basin inferred from GRACE satellite gravity data. Geophys. Res. Lett.

[b11] Dai A, Trenberth KE (2002). Estimates of freshwater discharge from continents: Latitudinal and seasonal variations. J. Hydrometeorol.

[b12] DeFries R, Hansen M, Townshend JRG, Janetos AC, Loveland TR (2000). A new global 1 km data set of percent tree cover derived from remote sensing. Global Change Biol.

[b13] Dooge JCI, Eagleson PS (1982). Parameterization of hydrological processes. Land Surface Processes in Atmospheric General Circulation Models.

[b14] Donohue RJ, Roderick ML, McVicar TR (2007). On the importance of including vegetation dynamics in Budyko’s hydrological model. Hydrol. Earth Syst. Sci.

[b15] Dunne KA, Willmott CJ (2000). 10.3334/ORNLDAAC/545.

[b16] Emery WJ, Thomson RE (2004). Data Analysis Methods in Physical Oceanography.

[b17] Eltahir EAB (1996). Role of vegetation in sustaining large-scale atmospheric circulations in the tropics. J. Geophys. Res.

[b18] Eltahir EAB, Bras RL (1994). Precipitation recycling in the Amazon basin. Q. J. R. Meteorol. Soc.

[b19] Eltahir EAB, Bras RL (1996). Precipitation recycling. Rev. Geophys.

[b20] Famiglietti JS, Wood EF (1994). Multi-scale modeling of spatially variable energy and water balance processes. Water Resour. Res.

[b21] Famiglietti JS, Wood EF (1995). Effects of spatial variability and scale on areally averaged evapotranspiration. Water Resour. Res.

[b22] Famiglietti JS, Lo M, Ho SL, Bethune J, Anderson KJ, Syed TH, Swenson SC, Rodell CR, De Linage M (2011). Satellites measure recent rates of groundwater depletion in California’s Central Valley. Geophys. Res. Lett.

[b23] Farmer D, Sivapalan M, Jothityangkoon C (2003). Climate, soil, and vegetation controls upon the variability of water balance in temperate and semiarid landscapes: Downward approach to water balance analysis. Water Resour. Res.

[b24] Gupta VK, Rodriguez-Iturbe I, Wood EF (1986). Scale Problems in Hydrology.

[b25] Kalnay E (1996). The NCEP/NCAR 40-year reanalysis project. Bull. Am. Meteorol. Soc.

[b26] Leblanc MJ, Tregoning P, Ramillien G, Tweed SO, Fakes A (2009). Basin-scale, integrated observations of the early 21st century multiyear drought in southeast Australia. Water Resour. Res.

[b27] Lettenmaier DP, Famiglietti JS (2006). Hydrology—Water from on high. Nature.

[b28] Liang X, Lettenmaier DP, Wood EF, Burges SJ (1994). A simple hydrologically based model of land surface water and energy fluxes for GSMs. J. Geophys. Res.

[b29] Makarieva AM, Gorshkov VG (2007). Biotic pump of atmospheric moisture as driver of the hydrological cycle on land. Hydrol. Earth Syst. Sci.

[b30] Makarieva AM, Gorshkov VG, Li B (2009). Precipitation on land versus distance from the ocean: Evidence for a forest pump of atmospheric moisture. Ecol. Complex.

[b31] McGuire KJ, McDonnell JJ, Weiler M, Kendall C, McGlynn BL, Welker JM, Seibert J (2005). The role of topography on catchment-scale water residence time. Water Resour. Res.

[b32] Milly PCD (1994). Climate, soil water storage, and the average annual water balance. Water Resour. Res.

[b33] Milly PC, Dunne KA (1994). Sensitivity of the global water cycle to the water-holding capacity of land. J. Clim.

[b34] Nash JE, Sutcliffe JV (1970). River flow forecasting through conceptual models. Part I: A discussion of principles. J. Hydrol.

[b35] Peel MC, McMahon TA, Finlayson BL (2010). Vegetation impact on mean annual evapotranspiration at a global catchment scale. Water Resour. Res.

[b36] Ramillien G, Famiglietti JS, Wahr J (2008). Detection of continental hydrology and glaciology signals from GRACE: A review. Surv. Geophys.

[b37] Reager JT, Famiglietti JS (2009). Global terrestrial water storage capacity and flood potential using GRACE. Geophys. Res. Lett.

[b38] Rodell M, Chen J, Kato H, Famiglietti JS, Nigro JD, Wilson CR (2007). Estimating groundwater storage changes in the Mississippi River basin (USA) using GRACE. Hydrogeol. J.

[b39] Rodell M, Velicogna I, Famiglietti J (2009). Satellite-based estimates of groundwater depletion in India. Nature.

[b40] Shukla J, Mintz Y (1982). Influence of land-surface evapotranspiration on the Earth’s climate. Science.

[b41] Spracklen DV, Arnold SR, Taylor CM (2012). Observations of increased tropical rainfall preceded by air passage over forests. Nature.

[b42] Swenson S, Wahr J (2002). Methods for inferring regional surface-mass anomalies from Gravity Recovery and Climate Experiment (GRACE) measurements of time-variable gravity. J. Geophys. Res.-Sol. Earth.

[b43] Swenson S, Wahr J (2006). Post-processing removal of correlated errors in GRACE data. Geophys. Res. Lett.

[b44] Swenson SC, Yeh PJ-F, Wahr J, Famiglietti JS (2006). A comparison of terrestrial water storage variations from GRACE with in situ measurements from Illinois. Geophys. Res. Lett.

[b45] Syed TH, Famiglietti JS, Zlotnicki V, Rodell M (2007). Contemporary estimates of Pan-Arctic freshwater discharge from GRACE and reanalysis. Geophys. Res. Lett.

[b46] Syed TH, Famiglietti JS, Rodell M, Chen J, Wilson CR (2008). Analysis of terrestrial water storage changes from GRACE and GLDAS. Water Resour. Res.

[b47] Tapley BD, Bettadpur S, Ries JC, Thompson PF, Watkins MM (2004). GRACE measurements of mass variability in the Earth system. Science.

[b48] Taylor KE, Stouffer RJ, Meehl GA (2012). An overview of CMIP5 and the experiment design. Bull. Am. Meteorol. Soc.

[b49] Trenberth KE, Fasullo JT, Mackaroo J (2011). Atmospheric moisture transports from ocean to land and global energy flows in reanalysis. J. Clim.

[b50] Ummenhofer CC, England MH, McIntosh PC, Meyers GA, Pook MJ, Risbey JS, Gupta AS, Taschetto AS (2009). What causes southeast Australia’s worst droughts?. Geophys. Res. Lett.

[b51] Verdin KL, Hall FG (2011). ISLSCP II HYDRO1k elevation-derived products. ISLSCP Initiative II Collection, Data set.

[b52] Wahr J, Swenson S, Zlotnicki V, Velicogna I (2004). Time-variable gravity from GRACE: First results. Geophys. Res. Lett.

[b53] Wahr J, Swenson S, Velicogna I (2006). Accuracy of GRACE mass estimates. Geophys. Res. Lett.

[b54] Yeh PJ-F, Famiglietti J, Swenson SC, Rodell M (2006). Remote sensing of groundwater storage changes using Gravity Recovery and Climate Experiment (GRACE). Water Resour. Res.

